# Beneficial Effects of Probiotic *Lactobacillus paraplantarum* BGCG11 on Pancreatic and Duodenum Function in Diabetic Rats

**DOI:** 10.3390/ijms25147697

**Published:** 2024-07-13

**Authors:** Mirjana Mihailović, Svetlana Soković Bajić, Jelena Arambašić Jovanović, Emilija Brdarić, Svetlana Dinić, Nevena Grdović, Aleksandra Uskoković, Jovana Rajić, Marija Đorđević, Maja Tolinački, Nataša Golić, Milica Živković, Melita Vidaković

**Affiliations:** 1 Institute for Biological Research “Siniša Stanković”, University of Belgrade, Bulevar Despota Stefana 142, 10060 Belgrade, Serbia; mista@ibiss.bg.ac.rs (M.M.); jelena.arambasic@ibiss.bg.ac.rs (J.A.J.); sdinic@ibiss.bg.ac.rs (S.D.); nevenag@ibiss.bg.ac.rs (N.G.); auskokovic@ibiss.bg.ac.rs (A.U.); jovana.rajic@ibiss.bg.ac.rs (J.R.); marija.sinadinivic@ibiss.bg.ac.rs (M.Đ.); 2 Institute of Molecular Genetics and Genetics Engineering, University of Belgrade, Vojvode Stepe 444a, P.O. Box 23, 11010 Belgrade, Serbiaemilija@imgge.bg.ac.rs (E.B.); maja_tolinacki@imgge.bg.ac.rs (M.T.); natasag@imgge.bg.ac.rs (N.G.)

**Keywords:** diabetes, glycohemoglobin, probiotic therapy, gut health, pancreas histology, zonulin, tight junction, microbiota

## Abstract

Diabetes mellitus, as a chronic metabolic disorder, significantly impacts the pancreas and among other organs, affects duodenal function. Emerging evidence suggests that probiotics can exert beneficial effects on gut health and metabolism. In our previous research, we evaluated the probiotic *Lactobacillus paraplantarum* BGCG11 primarily for its protective properties against diabetic rats’ damaged liver and kidneys. In this work, we further examined the effects of probiotic strain BGCG11 on the function of the duodenum and pancreas in diabetic rats. We explored the potential mechanisms underlying the probiotic’s effects, focusing on general indicators of diabetes, the architecture and morphology of pancreatic islets, duodenal integrity (measuring the transfer of fluid and serum zonulin level), and the modulation of gut microbiota composition. Our findings reveal the protective and regulatory roles of *L. paraplantarum* BGCG11 in mitigating diabetes-induced pancreatic and duodenal dysfunction regardless of its application time (pre- or post-treatment), highlighting its therapeutic potential in managing diabetes-related gastrointestinal complications.

## 1. Introduction

Diabetes is the most common metabolic disease with multifactorial origins, including genetic and environmental factors. Diabetes is characterized by hyperglycaemia, target-tissue resistance to insulin, and insufficient insulin secretion. The pancreas plays an important role in secreting insulin and glucagon hormones, which are essential for glucose/carbohydrate utilization and the regulation of blood glucose levels. Prolonged hyperglycaemia, increased oxidative stress, and inflammation promote different diabetic complications, such as neuropathy, nephropathy, retinopathy, macro and microvascular complications, and liver damage. In addition, about 75% of patients with diabetes have significant gastrointestinal disorders [1]. Gut microbiota modulates the functions of the gut immune system, affecting the innate and the adaptive immune systems. Immunological changes in the gut can profoundly affect the pancreas, modulating the incidence of diabetes as a result of the immunological link between the gut and pancreas [2,3]. Gastrointestinal problems are manifested through altered gut microbiota and increased intestinal permeability caused by changes in the tight junction. The consequence of the reduced expression of tight-junction proteins is increased endotoxins in the blood, metabolic endotoxaemia, an altered mucosal immune response, and insulin resistance, which contributes to the development of diabetes and diabetic complications [4,5]. However, the precise mechanisms that result in disorders such as leaky gut before the development of diabetes are still controversial and are not fully understood [6].

Gut microbiota is the major source of microbial stimulation that expresses both injurious and beneficial effects on homeostasis and health. Recent in-vitro and in-vivo studies have shown that adequate consumption of probiotics inhibits the growth of pathogenic bacteria, decreases the level of circulating endotoxins, reduces inflammatory responses, and stimulates the immune system [7,8]. *Bifidobacteria* and *Lactobacillus* are the most frequently used probiotic dietary supplements. It has been reported that dahi, a traditional Indian dairy product that contains *Lactobacillus acidophilus* and *Lactobacillus casei* probiotic strains, reduced HbA1c, lipid peroxidation, and oxidative stress in streptozotocin-induced diabetic rats [9]. Treatment with the *Bifidobacterium animalis* subsp. *lactis* 420 strain can reverse bacterial translocation from the intestine to tissues, thus preventing the early onset of high-fat diet-induced hyperglycaemia and leading to improved insulin sensitivity and equilibration/physiological balance between the inflammatory and metabolic status in a mouse model [10]. Several clinical studies on diabetic patients have also been undertaken. Studies in humans have shown that the gastrointestinal tract contains, on average, 10^14^ microorganisms/mL of luminal content and over 1000 different bacterial species [11]. Approximately 90% of the resident bacteria belong to the *Bacteroidetes* and *Firmicutes* phyla [12]. One study has shown that daily consumption of a symbiotic shake containing *Lactobacillus acidophilus*, *Bifidobacterium bifidum*, and oligofructose improved blood glucose concentration and led to a significant increase in HDL cholesterol [8,13]. Others showed that ingestion of probiotic yogurt containing *Lactobacillus acidophilus* La5 and *Bifidobacterium lactis* Bb12 for 6 weeks reduced fasting glucose, and HbA1c levels increased erythrocyte superoxide dismutase and glutathione peroxidase activities and the total antioxidant status in diabetic patients [14].

The probiotic strain *Lactobacillus paraplantarum* BGCG11 generates a “ropy” exopolysaccharide (EPS) and displays anti-inflammatory and immunosuppressive effects [15]. Previously, we showed that administration of the strain *Lactobacillus paraplantarum* BGCG11 had an antidiabetic effect and attenuated oxidative stress, inflammation, and fibrosis in the liver and kidneys of diabetic rats [16]. The current study’s objective was to evaluate the beneficial in-vivo effects of the strain *L. paraplantarum* BGCG11 on pancreatic and duodenal functionality in streptozotocin-induced diabetic rats.

## 2. Results

### 2.1. Administration of Probiotic L. paraplantarum BGCG11 Influences the Biochemical Parameters of Diabetes

The non-diabetic (ND) group of rats receiving the BGCG11 strain showed no signs of diarrhea or weight loss during the probiotic treatment. The body weight of both the ND group and non-diabetic group rats treated with probiotics (ND/P) displayed an increase in body weight of 30 ± 1.2%, indicating that the probiotic was not harmful. The diabetic group (D) exhibited a loss in body weight of 16.6 ± 0.6% at the end of the study when compared with the body weight at the start. The D group treated with probiotics displayed a loss of weight of 12 ± 0.4%. Food and water intake for the ND group and the ND/P group that received the probiotic were the same throughout the whole period of treatment, while in the D group, uptake of food and water was increased 4-fold for food and 10-fold for water when compared to the ND group. In the D groups treated with probiotics, food and water intake were lower as compared to the D group (2-fold for both food and water).

**Figure 1:**
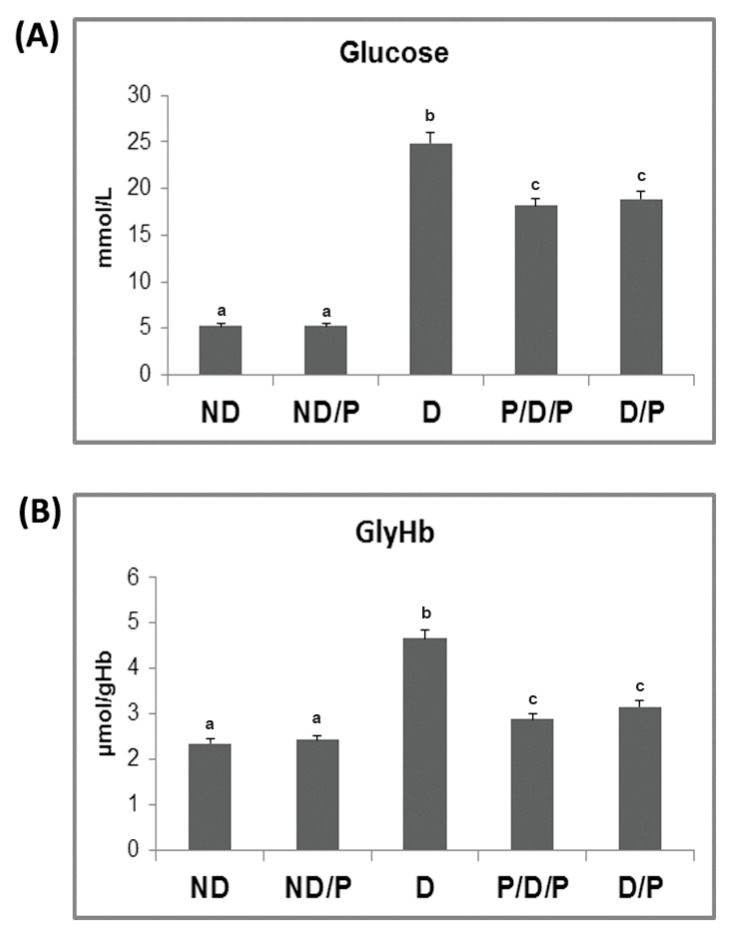
The effect of probiotic *Lactobacillus paraplantarum* BGCG11 administration on fasting blood glucose concentration (**A**) and level of glycated haemoglobin (**B**). ND—non-diabetic control group; ND/P—probiotic *Lactobacillus paraplantarum* BGCG11-treated non-diabetic group; D—streptozotocin (STZ)-induced diabetic group; P/D/P—one week pre-treatment and four weeks post-treatment with probiotic *Lactobacillus paraplantarum* BGCG11 of STZ-induced diabetic group; D/P—STZ-induced diabetic rats treated with probiotic *Lactobacillus paraplantarum* BGCG11 for four weeks. The values are means ± standard error of the mean (S.E.M.) from three separate measurements (technical replica). Groups’ means that do not have the same letter differ significantly from one another (*p* < 0.05).

The biochemical markers of diabetes, glucose, and glycohemoglobin (GlyHb) are presented in Figure 1. While both pre-treatment (P/D/P) (*p* < 0.05) and treatment (D/P) (*p* < 0.05) of D rats with the probiotic lowered the glucose concentration 3.5-fold, the concentration of glucose in diabetic rats was 4.7-fold higher (*p* < 0.05) than in the untreated ND rats (Figure 1A). In D rats, the level of GlyHb was 1.9-fold higher (*p* < 0.05) compared to the ND control (Figure 1B). GlyHb in the P/D/P group was 1.6-fold (*p* < 0.05) lower, while in the D/P group, the level of GlyHb was 1.4-fold (*p* < 0.05) lower when compared to the D group (Figure 1B).

### 2.2. Histological Changes, Collagen-Fibre Deposition, and Immunohistochemical Localization of Insulin and Glucagon in Pancreatic Islets Analysis after the Probiotic Administration

**Figure 2:**
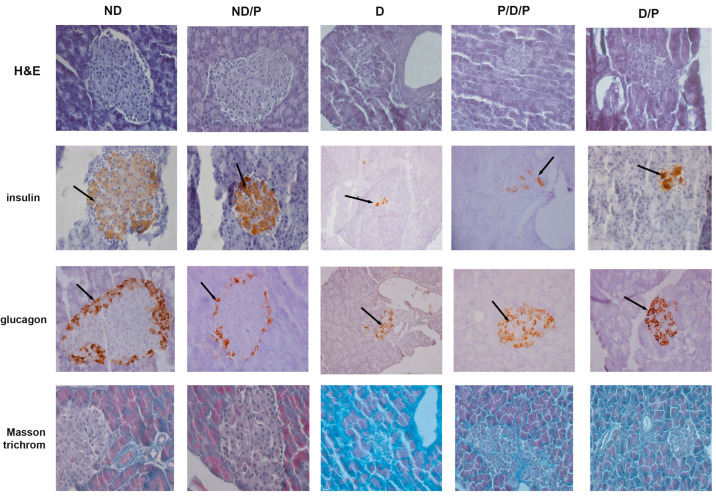
The effect of probiotic *Lactobacillus paraplantarum* BGCG11 administration on the histological changes, collagen-fibres deposition, and immunohistochemical localization of insulin and glucagon in pancreatic islets. H&E—hematoxylin and eosin staining of pancreatic sections; insulin—light photomicrographs of insulin immunohistochemical staining; glucagon—light photomicrographs of glucagon immunohistochemical staining; Masson trichrome—staining of pancreatic sections within islets (magnification 40×); ND—non-diabetic control group; ND/P—probiotic *Lactobacillus paraplantarum* BGCG11 treated non-diabetic group; D—STZ-induced diabetic group; P/D/P—one week pre-treatment and four weeks post-treatment with probiotic *Lactobacillus paraplantarum* BGCG11 of STZ-induced diabetic group; D/P—STZ-induced diabetic rats treated with probiotic *Lactobacillus paraplantarum* BGCG11 for four weeks. The black arrows indicate the insulin and glucagon accumulations.

H&E-stained sections from the control group showed that the pancreas had normal histology, consisting of closely packed lobules of pancreatic acini, while the islets of Langerhans consisted of numerous and compactly arranged cells that appeared as dense cords (Figure 2, ND, H&E). In the diabetic group, pathological changes in both exocrine and endocrine parts of the pancreas were revealed, manifesting vacuolation, smaller pancreatic islets, and a marked decrease in β-cells (Figure 2, D, H&E). In both P/D/P and D/P groups, improvement of cell architecture and morphology of pancreatic islets was observed; most of the islets of Langerhans were intact with only rare vacuoles (Figure 2, P/D/P and D/P, H&E). Treatment of control rats with the probiotics showed no differences compared to the control group (Figure 2, ND/P, H&E).

Insulin immunostaining in the control group showed a positive reaction, observed as brown granules in the cytoplasm in a considerable number of β-cells, with a homogeneous distribution (Figure 2, ND, insulin). In the diabetic group, the immunoreactivity for anti-insulin antibodies was defined in a small part of the islet as the consequence of an evident decrease in the number of insulin-positive cells (Figure 2, D, insulin) and islet atrophy. In both P/D/P and D/P groups, an increase in areas of the Langerhans islets with an increased number of positive immunoreactions of β-cells for anti-insulin antibodies was detected (Figure 2, P/D/P and D/P, insulin). The control pancreas of rats treated with the probiotic showed a strong insulin-positive reaction as observed in the control (Figure 2, ND/P, insulin).

Glucagon immunostaining in the control group was mostly observed along the islet periphery, as can be seen in the control group treated with the probiotic (Figure 2, ND, glucagon), where the majority of pancreatic α-cells are expected to be localised. In the diabetic group, an increase in glucagon-positive cells was observed; these cells were observed not only along the periphery but within the core of the islet (Figure 2, D, glucagon). In P/D/P and D/P groups, glucagon-positive cells were also found in the core of the pancreatic islet in a number that is much higher in comparison to the diabetic group (Figure 2, P/D/P and D/P, glucagon).

Positive Masson trichrome staining of the pancreas of control rats was observed in parts around cells of the islands of Langerhans, and slight collagen-fibre deposition was noted around the blood vessels and pancreatic acini (Figure 2, ND, Masson trichrome). In the diabetic group, Masson trichrome staining of the pancreas revealed thickening of the connective tissue septa with dense collagen fibres around the acini and blood vessels and that the fibrotic process of islets was advanced (Figure 2, D, Masson trichrome). In the P/D/P and D/P groups, collagen-fibre deposition and fewer regions of dense connective tissue septa were observed (Figure 2, P/D/P and D/P, Masson trichrome).

### 2.3. Effects of Probiotic Administration on Transfer of Fluid through the Intestinal Mucosa and the Serum Zonulin Level

**Figure 3:**
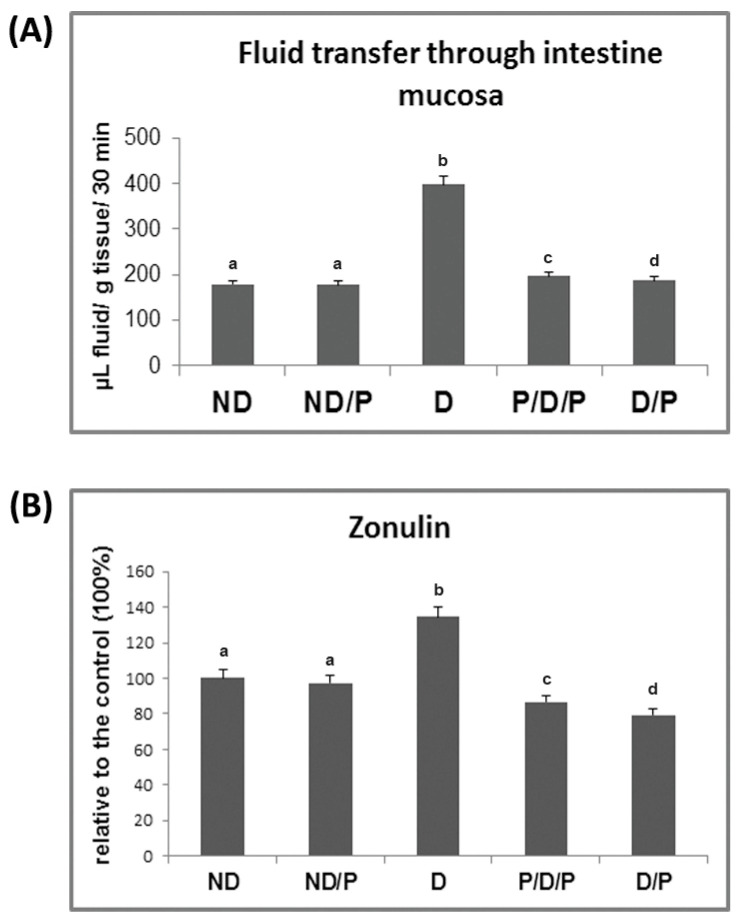
The effect of probiotic *L. paraplantarum* BGCG11 consumption on the transfer of fluid through intestinal mucosa (**A**) and serum zonulin levels (**B**). ND—non-diabetic control group; ND/P—probiotic *Lactobacillus paraplantarum* BGCG11 treated non-diabetic group; D—STZ-induced diabetic group; P/D/P—one week pre-treatment and four weeks post-treatment with probiotic Lactobacillus paraplantarum BGCG11 of STZ-induced diabetic group; D/P—STZ-induced diabetic rats treated with probiotic *Lactobacillus paraplantarum* BGCG11 for four weeks. Groups’ means that do not have the same letter differ significantly from one another (*p* < 0.05).

In the diabetic group, the transfer of fluid through the small intestinal mucosa is significantly more pronounced in comparison to the control animals (Figure 3A). Treatment with the probiotic in both analysed cases (P/D/P and D/P) led to a reduction in fluid transfer through the small intestinal mucosa (Figure 3A), which approached the control value.

As can be seen in Figure 3B, the concentration of serum zonulin increased 1.3-fold in the diabetic group when compared to the control group, while treatment with the probiotic decreased the serum levels of zonulin in both P/D/P and D/P rats. Treatment of control rats with the probiotic showed no difference in relation to the intact control group (Figure 3B).

### 2.4. Probiotic Administration Influences Histological Changes, Collagen-Fibre Deposition and Immunohistochemical Localization of Alpha-Smooth Muscle, Fibronectin and E-cadherin in the Duodenum Section

**Figure 4:**
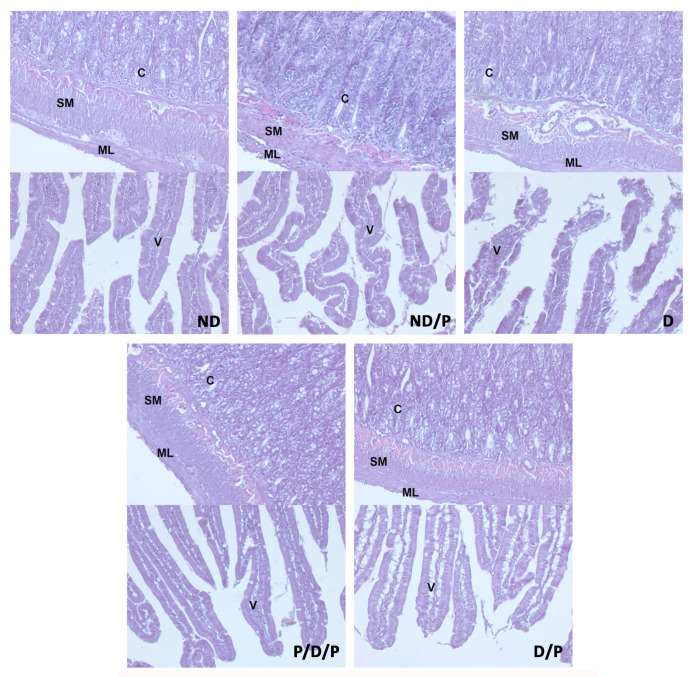
The effect of probiotic *Lactobacillus paraplantarum* BGCG11 administration on the histological changes of the intestine (duodenum section). H&E staining of duodenum section; V—villi (magnification 20×); SM—submucosa (magnification 40×); ND—non-diabetic control group; ND/P—probiotic *Lactobacillus paraplantarum* BGCG11 treated non-diabetic group; D—STZ-induced diabetic group; P/D/P—one week pre-treatment and four weeks post-treatment with probiotic *Lactobacillus paraplantarum* BGCG11 of STZ-induced diabetic group; D/P—STZ-induced diabetic rats treated with probiotic *Lactobacillus paraplantarum* BGCG11 for four weeks; C—crypts; SM—submucosa; ML—muscular layer; V—villi.

Histological examination of the duodenum in the control group revealed well-shaped villi with brush borders and crypts with clear lumen (Figure 4, ND). In the diabetic group, disorganisation and deformation of villi and intestinal glands were observed (Figure 4, D). In both P/D/P and D/P groups, decreased tissue damage and preservation of villi and intestinal glands were observed (Figure 4, P/D/P and D/P). Histological organisation in the ND/P group was similar to the control group (Figure 4, ND/P).

**Figure 5:**
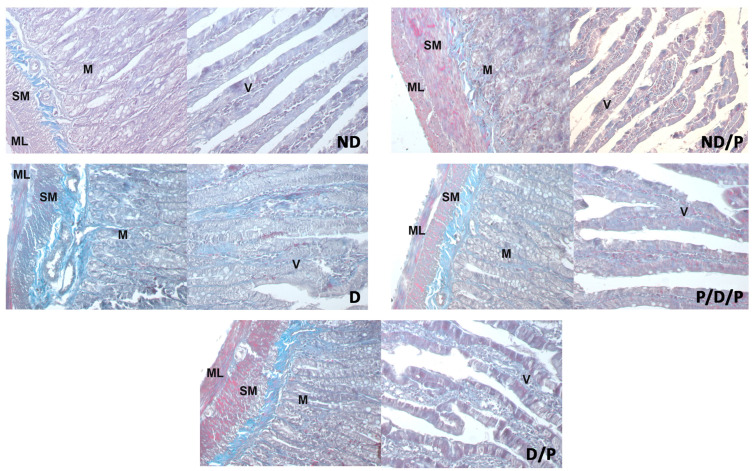
The effect of probiotic *Lactobacillus paraplantarum* BGCG11 administration on the collagen fibres deposition of the intestine (duodenum section). Masson trichrome staining of duodenum section; V—villi (magnification 20×); SM—submucosa (magnification 40×); ND—non-diabetic control group; ND/P—probiotic *Lactobacillus paraplantarum* BGCG11 treated non-diabetic group; D—STZ-induced diabetic group; P/D/P—one week pre-treatment and four weeks post-treatment with probiotic *Lactobacillus paraplantarum* BGCG11 of STZ-induced diabetic group; D/P—STZ-induced diabetic rats treated with probiotic *Lactobacillus paraplantarum* BGCG11 for four weeks; C—crypts; SM—submucosa; ML—muscular layer; V—villi.

Duodenal sections stained with Masson trichrome are shown in Figure 5. Sections from the control group showed delicate collagen fibres in the submucosa (Figure 5, ND), whereas in the diabetic group, an apparent increase in collagen fibres was observed throughout the muscular layer, the submucosa, and in the middle of the villus (Figure 5, D). Diminished collagen-fibre deposition was detected in sections from the duodenum of the P/D/P and D/P groups (Figure 5, P/D/P and D/P). In the duodenum section of the ND/P group, Masson trichrome staining showed similar collagen deposition as was observed in the control group (Figure 5, ND/P).

**Figure 6:**
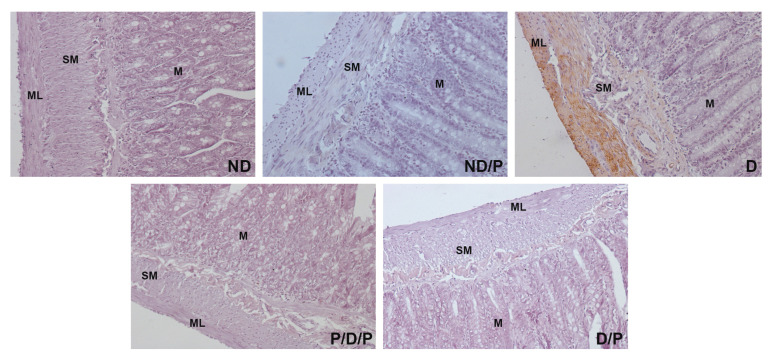
The effect of probiotic *Lactobacillus paraplantarum* BGCG11 administration on the immunohistochemical localization of alpha-smooth muscle actin in the intestine (duodenum section). Light photomicrographs of alpha-smooth muscle actin immunohistochemical staining of duodenum section (magnification 40×). ND—non-diabetic control group; ND/P—probiotic *Lactobacillus paraplantarum* BGCG11 treated non-diabetic group; D—STZ-induced diabetic group; P/D/P—one week pre-treatment and four weeks post-treatment with probiotic *Lactobacillus paraplantarum* BGCG11 of STZ-induced diabetic group; D/P—STZ-induced diabetic rats treated with probiotic *Lactobacillus paraplantarum* BGCG11 for four weeks; C—crypts; SM—submucosa; ML—muscular layer.

Alpha-smooth muscle actin (alpha-SMA) immunostaining was observed only in the muscular layer and submucosa of the duodenum section of the diabetic group (Figure 6, D), while in other experimental groups, no alpha-SMA-positive staining was detected (Figure 6, ND, ND/P, P/D/P, and D/P).

**Figure 7:**
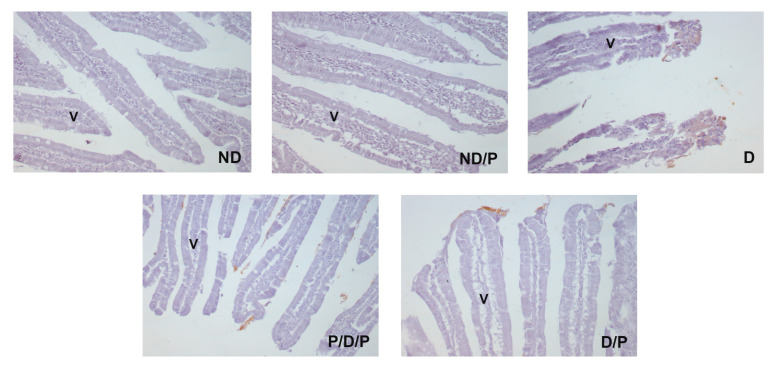
The effect of probiotic *Lactobacillus paraplantarum* BGCG11 administration on the immunohistochemical localization of fibronectin in the intestine (duodenum section). Light photomicrographs of fibronectin immunohistochemical staining of duodenum section (magnification 20×). ND—non-diabetic control group; ND/P—probiotic *Lactobacillus paraplantarum* BGCG11 treated non-diabetic group; D—STZ-induced diabetic group; P/D/P—one week pre-treatment and four weeks post-treatment with probiotic Lactobacillus paraplantarum BGCG11 of STZ-induced diabetic group; D/P—STZ-induced diabetic rats treated with probiotic *Lactobacillus paraplantarum* BGCG11 for four weeks; V—villi.

The marker of fibrosis, fibronectin-positive immunostaining, was observed in the upper part of the villi in the diabetic group (Figure 7, D), while in the P/D/P and D/P groups, decreased fibronectin-positive staining in the villi was observed (Figure 7, P/D/P and D/P). In the ND and ND/P groups, no fibronectin-positive staining was observed (Figure 7, ND and ND/P).

**Figure 8:**
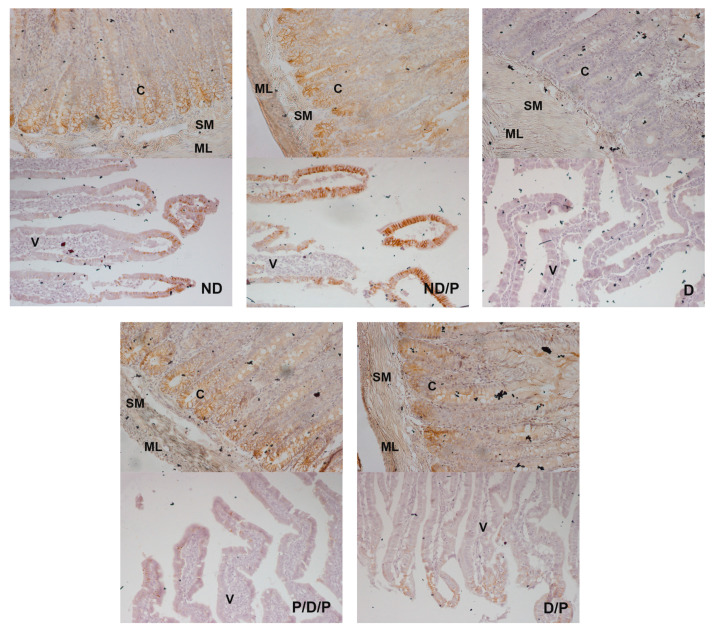
The effect of probiotic *Lactobacillus paraplantarum* BGCG11 administration on the immunohistochemical localization of E-cadherin in the intestine (duodenum section). Light photomicrographs of fibronectin immunohistochemical staining of duodenum section V—villi (magnification 20×); SM—submucosa (magnification 40×); ND—non-diabetic control group; ND/P—probiotic *Lactobacillus paraplantarum* BGCG11 treated non-diabetic group; D—STZ-induced diabetic group; P/D/P—one week pre-treatment and four weeks post-treatment with probiotic *Lactobacillus paraplantarum* BGCG11 of STZ-induced diabetic group; D/P—STZ-induced diabetic rats treated with probiotic *Lactobacillus paraplantarum* BGCG11 for four weeks; C—crypts; SM—submucosa; ML—muscular layer; V—villi.

E-cadherin (a prominent epithelial marker)-immunostaining was observed in the epithelium of the villi, crypts, the submucosa, and muscular layers in the duodenum sections in the ND and ND/P groups (Figure 8, ND and ND/P). In the diabetic group, no E-cadherin-positive staining was detected (Figure 8, D). In the P/D/P and D/P groups, E-cadherin immunostaining was observed in the same regions as in the control group but with a slightly weaker signal, especially in the villi part (Figure 8, P/D/P and D/P).

### 2.5. Effects of Probiotic BGCG11 Administration on Changes in Gut Microbiota Composition

We profiled the gut microbiota composition in each experimental group and identified several associations between bacterial taxa and metabolic pathways. We compared the final effect of the treatment of diabetic rats with probiotic BGCG11 on changes in gut microbiota in the case when the disease was induced at the same time as the initiation of probiotics in the D/P group and rats in which diabetes was induced during the administration of probiotics in the P/D/P group with the diabetic group of rats. There were no significant changes in the alpha diversity during the whole experiment (the beginning, induction, and the end of treatment) in the D group or in the P/D/P group, as well as in treatment (D/P in induction and at the end), and also no significant changes were observed at the end of the experiment for all groups (Figure S1).

Regarding beta diversity, notable different clustering of groups was observed in the D group and the P/D/P group during the experiment (the beginning, induction, and the end of treatment), while in the treatment (D/P induction and D/P end) and at the end for all groups, no such clustering of groups was observed (Figure S2).

**Figure 9:**
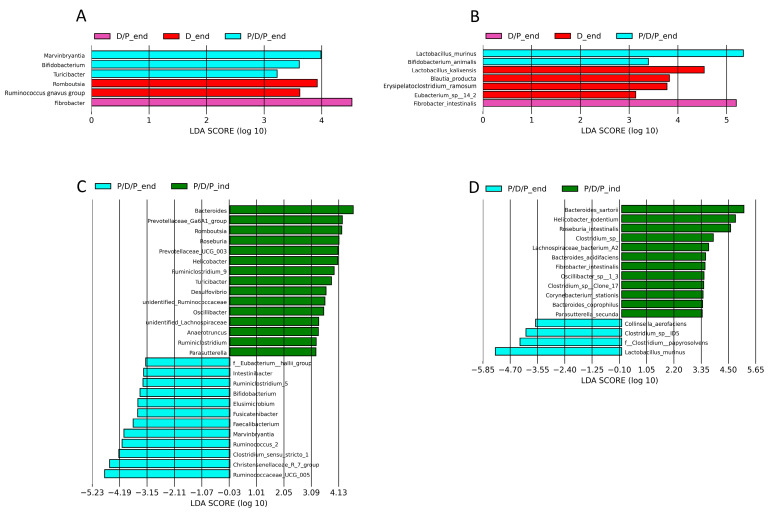
Graphical display of genus and species among groups given by Linear discriminant analysis effect size (LEfSe) of gut microbiota at genus level at the end of the experiment in all groups (**A**) and for the same groups at the species level (**B**), and between induction of diabetes and the end of the experiment for the P/D/P group at the genus level genus (**C**) and at the species level (**D**).

To compare the relative abundances between the groups, we conducted a linear discriminant analysis effect size (LEfSe), as shown in Figure 9. Genera *Marvinbryantia, Bifidobacterium, Lactococcus*, and *Turicibacter*, as well as species *Bifidobacterium animalis* and *Lactobacillus murinus*, were shown to be microbial markers of the P/D/P group, whereas *Fibrobacter intestinalis* were enriched in the D/P group, and genera *Romboutsia* and *Ruminococcus gnavus*, as well as species *Lactobacillus kalixensis, Blautia producta, Erysipelatoclostridium ramosum*, and *Eubacterium* sp. were enriched in the D group (Figure 9A,B).

During the moment of diabetes induction, LEfSe analysis revealed that 15 bacterial genera (*Bacteroides*, *Prevotellaceae*_Ga6A1_group, *Romboutsia*, *Roseburia*, *Prevotellaceae*_UCG_003, *Helicobacter*, *Ruminiclostridium*_9, *Turicibacter*, *Desulfovibrio*, Unidentified*_Ruminococcaceae*, *Oscillibacter*, Unidentified_*Lachnospiraceae*, *Anaerotruncus*, *Ruminiclostridium*, and *Parasutterella* (Figure 9C) and 12 bacterial species (*Bacteroides sartorii*, *Helicobacter rodentium*, *Roseburia intestinalis*, *Clostridium* sp., *Lachnospiraceae* bacterium A2, *Bacteroides acidifaciens*, *Fibrobacter intestinalis*, *Oscillibacter* sp. 1_3, *Clostridium* sp. Clone 17, *Corynebacterium stationis*, *Bacteroides coprophilus*, and *Parasutterella secunda*) were enriched in the P/D/P group (Figure 9D). After four weeks of treatment with BGCG11, the enrichment pattern changed. Twelve bacterial genera (*F_Eubacterium hallii*_group, *Intestinibacter*, *Ruminiclostridium_*9, *Bifidobacterium*, *Elusimicrobium*, *Fusicatenibacter*, *Faecalibacterium*, *Marvinbryantia, Ruminococcus_2 Clostridium* sensu stricto 1, *Christensenellaceae*_R7_group, and *Ruminococcaceae* UCG 005) and four bacterial species (*Collinsella aerofaciens*, *Clostridium sp. ID5*, *Clostridium papyrosolvens*, and *Lactobacillus murinus*) were enriched at the end of the treatment period (Figure 9C,D).

**Figure 10:**
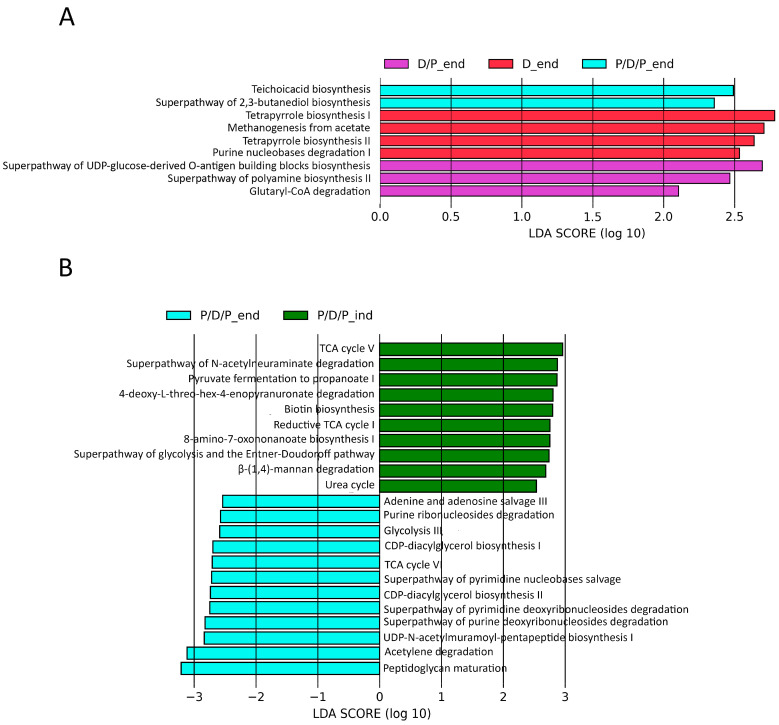
Differentially abundant predicted metabolic pathways generated using the PICRUSt2 pipeline with default options, showing discriminative predicted pathways among the groups at the end of the experiment (**A**) and for the P/D/P group at the moment of diabetes induction and at the end of the experiment (**B**).

Furthermore, we used the PICRUSt2 pipeline with default options to compare projected microbial metabolic functions between the groups in order to evaluate probable microbial functional composition, presenting only pathways that reached an LDA significance threshold of >2.5. At the end of the treatment, these analyses revealed several pathways involved in teichoic acid biosynthesis (TEICHOICACID-PWY) and butanediol biosynthesis (PWY-6396) as potential markers of the P/D/P group. The D/P group was characterized by pathways involved in lipopolysaccharide (PWY-7328) and polyamines (POLYAMINSYN3-PWY) biosynthesis and carboxylate degradation (PWY-5177). According to the findings, the D group was distinguished by an enrichment of the pathways involved in methanogenesis from acetate (METH-ACETATE-PWY), tetrapyrrole biosynthesis (PWY-5188, PWY-5189), and purine nucleotide degradation (P164-PWY) (Figure 10A).

We also compared which metabolic pathways were enriched during the continuous administration of probiotics at the end of the treatment, P/D/P_end, with those that were enriched during the time of diabetes induction, P/D/P_ind. The analyses indicated that at the time of disease induction, the P/D/P group was marked by enrichment pathways involved in biotin (BIOTIN-BIOSYNTHESIS-PWY, PWY-6519) biosynthesis, precursor metabolites, and energy generation (PWY-6969, GLYCOLYSIS-E-D, P108-PWY), CO_2_ fixation reductive (P23-PWY), carboxylate degradation (P441-PWY), carbohydrate degradation (PWY-6507, PWY-7456), and urea cycle (PWY-4984). At the end of the treatment, the P/D/P group displayed the greatest quantity of enriched pathways implicated in purine and pyrimidine nucleotide salvage (PWY-6609, PWY-7208) and degradation (PWY0-1296, PWY0-1297, PWY0-1298), generation of precursor metabolites and energy (ANAGLYCOLYSIS-PWY, PWY-6969, P161-PWY), phospholipid biosynthesis (PWY-5667, PWY0-1319), and peptidoglycan biosynthesis (PWY-6387, PWY0-1586) (Figure 10B).

## 3. Discussion

Intestinal dysfunction has been intensively studied in the aetiology of diabetes [17,18,19]. It has been shown that disturbed resident gut microbiota is associated with chronic inflammation and that it contributes to diabetes onset [20]. Altered gut microbiota and inflammation lead to increased intestinal permeability and metabolic endotoxaemia [2,4]. A connection between the microbiome and pathogenesis of type-2 diabetes (T2D) and impaired glucose tolerance has been reported, as well as in the pathogenesis of type-1 diabetes (T1D) and disturbed glycaemic response [21]. Altered gut microbiota and inflammation also lead to disturbed normal glucose tolerance, insulin resistance, modulation of gut peptide YY, and glucagon-like peptide-1 secretion [22]. A review by Kobyliak et al. elaborated on the efficacy of probiotics for the protection of pancreatic beta cell damage and explained the mechanism of probiotic action in patients with T2D showing amelioration of diabetes [23]. Probiotics are live microorganisms naturally occurring in fermented food products with multiple health benefits, including in diabetes and diabetic complications [24,25]. *Bifidobacterium* sp. and *Lactobacillus acidophilus* are most commonly used by humans and have been investigated for their potential effect on human health [26]. *L. paraplantarum* BGCG11 was isolated from soft, white, homemade cheese. It produces EPS as a heteropolysaccharide, showing a “ropy” phenotype, denoted by the formation of a long filament in the culture, which is due to the synthesis of EPS. The biopolymer is a heteropolysaccharide mainly composed of glucose (76%) and rhamnose (21%) with some traces of galactose [27]. In previous articles, we have shown that *L. paraplantarum* BGCG11 was able to survive simulated gastrointestinal transit (GIT) and was able to elicit different in-vitro immune responses upon peripheral blood mononuclear cells; it has an anti-inflammatory or immunosuppressor profile, thus could be included in the diet of patients suffering diseases associated with an increased inflammatory status [15]. In addition, purified EPS-CG11 synthesized by the strain *L. paraplantarum* BGCG11 was able to partially counteract the HT29-MTX cellular damage (release of LDH) caused by some pathogens [28].

In that regard, *L. paraplantarum* BGCG11 was chosen for evaluation of its probiotic potential in diabetic rats. We showed that both pre- and post-treatment with the probiotic BGCG11 showed an antidiabetic effect in STZ-induced diabetic rats. We observed that administration of this probiotic improved the disturbance in redox homeostasis, inflammation, and fibrosis in the liver and kidneys of diabetic rats, and as a result of the improvement of the antioxidant system, an increase in E-cadherin and decrease in α-smooth muscle actin and fibronectin [16]. In this paper, we have further analysed its protective mechanism in diabetes. The study’s findings demonstrate that giving diabetic rats the BGCG11 strain orally both before and after treatments enhanced the histology of the pancreatic islets, the areas that produce insulin, and decreased collagen-fibre deposition as a marker of fibrosis. After both treatments, glucagon-positive cells were found in the core of the pancreatic islet, which was obviously a reflection of the diabetes-related destruction of pancreatic morphology linked to impaired glucagon signalling that leads to a marked increase in alpha cell mass [29]. Although a different animal model was used in comparison to our research, it is interesting that in several studies, different lactobacilli exhibited protective effects on diabetic animals. For example, *Lactobacillus paracasei* strain NL41 was able to prevent induced diabetes (HFD/STZ-T2DM) in rats by reducing insulin resistance and oxidative-stress status and protecting beta-cell function [30]. In HFD and type-2 diabetic models, *Lactobacillus fermentum* MCC2759 and MCC2760 showed beneficial effects through increased GLUT4, GLP1, and adiponectin expression, improved intestinal barrier function, and decreased pro-inflammatory cytokines in the liver, intestine, MAT, and muscle tissue [31]. *L. plantarum* HAC01 could alleviate hyperglycaemia and T2DM by regulating glucose metabolism in the liver, protecting the islet β-cell mass, and restoring the gut microbiota and SCFAs in mice [32]. Moreover, in mice, *L. plantarum* Pro1 and a combination of *L. rhamnosus* Pro2 and *L. plantarum* Pro1 reduced the harmful effects of high levels of fructose in the blood, repaired histopathological changes in the pancreas, and altered the expression of GH, IGF1, and GLP-1 genes to normal levels, which are important for a balance between pancreatic islet cell proliferation and apoptosis [33]. Regarding the effect of our BGCG11 strain, its treatment decreased the disturbance in intestine permeability (decrease in zonulin secretion, improvement of fluid transfer through the intestinal mucosa), improved intestinal histology, and decreased the fibrotic process (lowered collagen-fibre deposition, cytoskeletal marker alpha-SMA and extracellular protein fibronectin, and increased cell-surface marker, E cadherin).

Endotoxaemia in T1D induces oxidative stress in the pancreas, destroys β-cells, and impairs insulin production [8]. The results of this study revealed the destruction of pancreatic islets in diabetic rats, whereas the islets were preserved after both pre- and posttreatment with the probiotic. In accordance with this result, we also observed collagen-fibre deposition in the islets of diabetic rats, which was noticeably reduced in the pancreatic islet in both probiotic-treated groups. Preserved structure and reduced fibrosis in pancreatic tissue in the probiotic-treated groups were followed by increased functionality, reflected in increased areas of the pancreatic islets that produce insulin. The possible mechanism underlying this positive effect of the probiotic on the pancreas could be the result of the attenuation of inflammation and endotoxaemia observed in diabetes. In diabetes, altered proportions of bacterial phyla in the intestine and consequent endotoxaemia increase the absorption of lipopolysaccharide, which spreads in circulation, initiating activation of inflammatory pathways and impairment of insulin signalling [34]. For example, pancreatic immunity and disease pathogenesis are significantly influenced by the gut–pancreatic axis, which is regulated by the microbiome. There is a growing amount of evidence supporting the role of the intestinal microbiota in pancreatic disease diagnosis and treatment. Faecal microbiota transplantation (FMT) is therefore becoming recognized as a potentially effective treatment option for a number of pancreatic conditions, such as cancer, pancreatitis, and type-1 diabetes (T1D) [35]. Recent evidence indicates that transformed gut microbiota contribute to the different changes observed in diabetes and are thus of great interest as potential treatments for obesity and diabetes. Histological evidence for pancreatic islet destruction in BioBreeding diabetic-prone rats indicates that increased intestinal permeability occurs at least one month before manifest diabetes [36]. Some intestinal aberrations in human autoimmune diabetes have been also reported. In patients with T1D, increased intestinal permeability, ultrastructural changes, and increased circulating zonulin have been observed [37]. Increased permeability of the intestinal epithelium could enable contact with food antigens, enteroviruses, live bacteria, and bacterial products with the mucosal immune system leading to intestinal inflammation, and it could also trigger T1D [38]. Blockage of the cannabinoid receptor-1 (CB1) using CB1 antagonists lowered gut permeability in obese mice through normalizing occludin and zonulin expression [39]. In accordance with the literature data, the results of this study show the ameliorating effect of the probiotic *Lactobacillus paraplantarum* BGCG11 on the reduction in fluid transfer through the small intestinal mucosa. In patients with diabetes, as in animals with experimental-induced diabetes, disorders occur in a thin permeability hose at different levels, including the transfer of fluid and glucose through the intestinal mucosa. Potential mechanisms that underlie the verified effects of probiotics (including the effects of *L. paraplantarum* BGCG11) on diabetes may include changes in transport capacity, passive permeability, and changes in the mucous membrane of the small intestine. All mentioned diverse effects require more detailed investigation and experimental proof. The integrity of the intestinal epithelium is primarily preserved by intercellular junctional complexes such as tight junctions (TJs). Circulating zonulin, a pre-haptoglobin molecule that has, up to now, been only regarded as the inactive precursor of haptoglobin, is set as a marker of intestinal permeability in autoimmune diseases and diabetes [40,41]. By modulating intercellular tight junctions, zonulin can influence intestinal permeability [42]. Recently, zonulin was detected in an uncleaved form in human serum, and a major biological effect of zonulin in influencing the integrity of intercellular TJ was proposed [43]. Several potential intestinal luminal stimuli can trigger zonulin release, and when intestinal permeability is disrupted, serum zonulin levels are increased. It has been reported that abnormal serum zonulin levels are associated with increased intestinal permeability in a subgroup of T1D patients [42]. In BioBreeding diabetes-prone rats, the zonulin-dependent increase in intestinal permeability was recorded 2–3 weeks before the onset of T1D [44]. Multi-strain probiotic formulation (*L. rhamnosus*, *B. lactis*, and *B. longum*) modulates the expression of TJ proteins and prevents inflammatory damage [45]. The results reported in this study show that the probiotic strain *L. paraplantarum* BGCG11 significantly reduced serum zonulin levels after pre- and post-treatment of diabetic rats.

The observed decrease in fluid transfer through small intestine mucosa and serum zonulin levels in diabetic rats treated with the probiotic correlates with other results obtained in this study. Increased collagen-fibre deposition observed in the duodenal section through the muscular layer, the submucosa, and in the middle of the villus was significantly decreased after treatment with the probiotic. In line with collagen deposition, the diabetic state was also accompanied by the increased presence of two fibrotic marker proteins. Tissue fibrosis is characterized by the deposition of fibronectin, the extracellular matrix glycoprotein, which serves as a scaffold for collagen and other extracellular matrix proteins [46]. Also, the de novo appearance of alpha-SMA in response to tissue injury and its increased expression is frequently used as a marker of fibrosis [47]. The levels of both fibrotic markers were markedly decreased in diabetic rats treated with the probiotic when compared to untreated diabetic animals. The antifibrotic effects of the probiotic were additionally confirmed by the finding that the expression of the epithelial cell marker E-cadherin remained elevated in probiotic-treated rats, while complete loss of its expression was observed in diabetic rats as another hallmark of fibrosis development.

The full mechanism of probiotic action in diabetes is still unclear. Higher excretion of glucagon-like peptide 1 (GLP-1) from intestinal L-cells, which raises insulin sensitivity, is one of the recognized mechanisms of probiotic activity [9,48,49]. For example, the genetically engineered strain *Lactobacillus plantarum* (*L. plantarum*)-pMG36e-GLP-1 regulated the intestinal microbiota, the apoptosis of pancreatic islet cells was inhibited, islet β-cell proliferation and insulin secretion were all promoted, and it showed its ameliorative effect on T2DM in artificially induced mice and transgenic mice [50]. Clinical trials in humans revealed, using the HOMA2 calculator, that probiotic therapies have a slight positive effect on the function of β-cells in individuals with T2D [51]. Other points of action of probiotics in diabetes are linked with an improvement in intestinal integrity, diminishment of endotoxaemia, and immunomodulatory, anti-inflammatory, and antioxidant effects [2,16,52]. Recently it was shown that *Lacticaseibacillus paracasei* SD1 and *Lacticaseibacillus rhamnosus* SD11 attenuate inflammation and β-cell death in streptozotocin-induced type-1 diabetic mice, preserve islet integrity and increased β-cell mass, upregulate anti-apoptotic Bcl2, as well as prevent infiltration of macrophages, CD4+, and CD8+ T cells into the islets [53]. Another study has shown that for T2DM mice, supplementing with the *Bifidobacterium longum* NBM7-1 and *Lactiplantibacillus rhamnosus* NBM17-4 alleviates glucose intolerance through the upregulation of IL-22, which enhances insulin sensitivity and pancreatic functions while reducing liver steatosis [54]. In light of the findings presented herein, we can conclude that enhancement of the epithelial barrier and a decrease in intestinal permeability are essential elements of the *L. paraplantarum* BGCG11 probiotic’s mode of action, as well as the lowering of the fibrotic process, which leads to a reduction in endotoxin concentrations in circulation and its adverse consequences.

It was observed that unlike the use of probiotic BGCG11 strain after the onset of diabetes, continuous application of the probiotic leads to a statistically significantly increased abundance of genera *Marvinbryantia, Bifidobacterium,* and *Turicibacter*, as well as species *Lactobacillus murinus* and *Bifidobacterium animalis,* taxa associated with enhanced insulin sensitivity, and the production of short-chain fatty acids (SCFA). The literature data indicate that butyric acid enhances insulin sensitivity, promotes pancreatic insulin production, and modifies insulin signaling [55]. It has significant functions, such as providing anti-obesity effects, reducing metabolic stress, and inhibiting inflammatory reactions [56]. *Marvinbryantia* butyrate-producing bacteria may reduce the incidences of insulin resistance, and these genera were more prevalent in healthy controls than in T2D [57,58]. Several studies have found that *Bifidobacterium* sp. can improve insulin sensitivity in people with T2D. In high-fat diet mice, administration of *B. animalis* ssp. lactis increased insulin sensitivity, decreased inflammatory cytokine levels, and ameliorated T2DM liver injuries [59,60]. Likewise, a combination of the two strains *Bifdobacterium* BL21 and *Lacticaseibacillus LRa05* can ameliorate T2DM in mice by regulating the intestinal flora and reducing inflammation and oxidative stress [61]. *Turicibacter* has also been associated with butyric acid production in the intestine [62]. Diabetes is known to be a risk factor for various complications, including the development of mesenteric ischemia, and one study has demonstrated that *Lactobacillus murinus* can help mitigate the negative effects of intestinal ischemia/reperfusion injury [63,64].

Analysis of the obtained results revealed that continuous probiotic treatment had a better effect on the microbiota of diabetic rats, so we decided to compare the changes in the microbiota between the end of treatment in the P/D/P group (P/D/P_end) and the time of disease induction (P/D/P_ind). During a maintained application of the probiotic in the P/D/P group, we saw numerous shifts in relative abundances at various taxonomic levels. At the end of treatment, a reduction in potentially harmful bacteria was observed. A high relative abundance of bacterial taxa, negatively correlated with diabetic complications, was observed during disease induction in the P/D/P_ind group but not at the end of the treatment for the P/D/P_end group, such as genera *Helicobacter*, *Ruminococcaceae*, *Oscillibacter Lachnospiraceae*, and *Parasutterella*, and species *Lachnospiraceae bacterium,* and *Corynebacterium stationis.* This is consistent with prior data revealing that *Ruminococcaceae* was the predominant family in T1D and that *Oscillibacter*, an opportunistic pathogen, was associated with T1D, T2D, and obesity in humans and mice [65,66]. Additionally, research indicates that intestinal colonization by a *Lachnospiraceae bacterium* contributes to the development of diabetes in obese mice [67]. Furthermore, *Parasutterella* was positively associated with BMI and T2D, and *Corynebacterium stationis* bacteria correlated with diabetic foot infections [68,69].

The composition of the gut microbiota continued to evolve under the influence of the probiotic treatment, and LEfSe analysis has shown that the intestinally beneficial bacteria were significantly more prevalent at the end of treatment. Among the beneficial bacteria, we identified the presence of bacterial taxa that negatively correlate with diabetes, such as *Intestinibacter*, *Bifidobacterium*, *Elusimicrobium*, *Fusicatenibacter*, *Faecalibacterium*, *Marvinbryantia*, *Clostridium sensu stricto* 1, *Christensenellaceae_*R7_group, and *Ruminococcaceae* UCG 005. One study showed that a higher abundance of *Christensenellaceae*, *Clostridiaceae* 1, *Peptostreptococcaceae*, *Christensenellaceae* R7 group, *Marvinbryantia*, *Ruminococcaceae* UCG005, *Ruminococcaceae* UCG008, *Ruminococcaceae* UCG010, *Ruminococcaceae* NK4A214 group, *C sensu stricto* 1, *Intestinibacter*, and *Romboutsia* may benefit the risk of insulin resistance and T2D [57]. Probiotic *L. paracasei* NL41 induced changes in the microbiota structure; for example, *Bacteroides*, *Clostridia* (specifically, *Ruminococcus torques*), and *Parasutterella* were significantly reduced, while some beneficial microorganisms (Bacteroidales_S24-7_group and the families *Lachnospiraceae* and *Ruminococcaceae*) were enriched by NL41 [70]. Other studies revealed that the genera of *Bifidobacterium*, *Bacteroides*, *Faecalibacterium*, *Akkermansia*, and *Roseburia* were negatively associated with T2D. Also, bacterial genera *Faecalibacterium, Fusobacterium, Dialister*, *Elusimicrobium*, *Anaerostipes*, *Coprococcus*, *Fusicatenibacter*, *Lachnospira*, *Marvinbryantia*, *Roseburia*, *Faecalibacterium*, *Ruminococcus*, *Subdoligranulum*, UCG-002, *Agathobacter*, *Butyricicoccus*, *Alistipes*, *Clostridium sensu stricto* 1, and all *Lachnospiraceae* were identified in significantly higher abundance in healthy individuals than in people with diabetes [71,72,73].

The literature data revealed that a *Ruminococcaceae* UCG-005 abundance positively correlated with SCFA concentration in the faeces of piglets, and *Intestinibacter bartlettii* produces the SCFAs isobutyrate and isovalerate, while *Marvinbryantia* and *Clostridium sensu stricto* 1 were butyrate-producing bacteria [74,75,76,77]. Furthermore, *Bifidobacterium* species have been proven to lower serum glucose levels, increase insulin-signaling protein expression, and improve adipokine profiles in diabetic mice [78]. It is a very significant finding that under diabetic conditions, *Faecalibacterium prausnitzii* anti-inflammatory compounds could restore the gut barrier and zonula occludens-1 expression, possibly through the tight junction pathway [79]. Also, Mancabelli et al. reported *Christensenellaceae* as one of five taxa considered a signature of a healthy gut [80].

Because 2, 3-butanediol has been found to be effective in the treatment of conditions such as obesity, insulin resistance, and diabetes, the enrichment of the pathway leading to butanediol biosynthesis (PWY-6396) was identified as a potential marker pathway in the P/D/P_end group (WO2021038253A1WIPO (PCT)) (https://patents.google.com/patent/WO2021038253A1/en (accessed on 14 July 2023)). On the other hand, the super pathway of polyamine biosynthesis II (POLYAMINSYN3-PWY) could be selected as a potential marker pathway in the D/P_end group. Polyamines, such as spermine, may have a role in maintaining mitochondrial energetics in a high-glucose environment. Moreover, supplementation of spermine improves glucose uptake, reduces myocardial cell death and oxidative stress, and attenuates cardiac dysfunction in the streptozotocin-induced T1D hearts [81]. Our findings suggest that a putative marker pathway in the D_end group could be a process involved in methanogenesis (METH-ACETATE-PWY). A recent study revealed that changes in intestinal methane production play a role in diabetes glycemic management [82]. In diabetic individuals, intestinal methane production is additionally related to reduced glucose tolerance and may be linked to altered glycemic control [83].

Different TCA and glycolysis pathways, as well as pathways involved in biotin biosynthesis on one side, pathways involved in purine and pyrimidine nucleotide biosynthesis and degradation, pathways involved in the synthesis of acetate and lactate (glycolysis III and pyruvate fermentation to acetate and lactate II) and phospholipid and peptidoglycan biosynthesis were shown as potentially dominant microbial marker pathways in the P/D/P_ind and P/D/P_end groups, respectively. In T1D, the TCA cycle is known to be significantly altered, which contributes to the development of cardiovascular autonomic neuropathy [84]. Similar findings, including an inefficient mitochondrial TCA cycle influx and reduced energy yield in skeletal muscle, were observed in T2D [85]. Also, using biotin as an adjuvant to the insulin regimen has been shown to improve glycemic control and lower plasma lipid concentrations in poorly managed T1D patients [86]. Also, it has been demonstrated that, in addition to altering insulin secretion in pancreatic cells, phospholipids can alter the effect of insulin in a variety of physiological processes [87].

In summary, diabetes-related gut health is a complex and interconnected system involving the gut microbiota, gut barrier integrity, inflammation, and metabolic regulation. The strain BGCG11 was able to assist in the reparation and restoration of both the pancreas and intestines of diabetic rats while assembling the beneficial microbiota. Ongoing research continues to unravel the intricate mechanisms underlying these relationships, paving the way for innovative therapeutic approaches to improve gut health and overall metabolic outcomes in individuals with diabetes.

## 4. Materials and Methods

### 4.1. Preparation of Bacteria

The study used *Lactobacillus paraplantarum* BGCG11, a naturally occurring isolate from soft, white, handmade cheese from Serbia. Bacteria were cultivated in De Man-Rogosa-Sharpe (MRS) medium (Merck GmbH, Darmstadt, Germany) at 30 °C. For the animal treatment, 10 mL of overnight culture (containing in total 2 × 10^8^ to 3 × 10^8^ CFU/mL) was pelleted, and the bacterial precipitate was washed in saline and resuspended in 1 mL of 11% sterile skimmed milk (AD Mlekara Subotica, Subotica, Serbia), as described by Geier et al. [88].

### 4.2. Animals

Experiments were performed on 2.5-month-old adult albino Wistar rats weighing 220–250 g. All animal procedures complied with Directive 2010/63/EU on the protection of animals used for experimental and other scientific purposes. The procedures were approved by the Ethical Committee for the Use of Laboratory Animals of the Institute for Biological Research “Siniša Stanković”, University of Belgrade, Serbia, No. 02-12/14.

### 4.3. Experimental Protocol

The animals were fasted overnight and sacrificed by euthanasia using a guillotine, according to all recommendations of the guidelines for euthanasia of animals. Death comes quickly without any suffering for the animals. Euthanasia was performed on animals one by one (in the block separated from the experimental room where the animals were held in order to avoid stress to the animals) by well-trained staff. Immediately before sacrifice, blood was collected from the tail vein of the animal for glucose measurement and for the preparation of serum, which was stored at −20 °C until use. After blood collection and sacrifice, an abdominal incision was made, and the tail part of the pancreas was removed and fixed in 10%-buffered formalin for histological examinations. The pancreas and duodenum from all experimental groups were removed and fixed in 10%-buffered formalin for histological and immunohistological examinations.

STZ (MLDS)-induced diabetes was employed as the experimental model. Male albino Wistar rats were given an intraperitoneal injection of STZ (40 mg/kg/day) (MP Biomedicals, Solon, OH, USA) for five days in order to induce diabetes. Prior to the injection, STZ was dissolved in a sodium citrate buffer (0.1 M, pH 4.5). Twenty-four hours following the last STZ injection, blood glucose was evaluated. Rats that had fasted for the whole night had blood samples taken from their tail veins, and a blood glucose meter (Accu-Check Active, Roche Diagnostics Scientific Office, Cairo, Egypt) was used to determine the glucose levels. When the blood glucose level in rats was more than 20 mmol/L while fasting, the rats were diagnosed as diabetic. The rats were split into the following five groups at random: (i) ND—the non-diabetic group (n = 8), or the negative control, that were given injections of citrate buffer equivalent to those of STZ for five days in a row; (ii) ND/P—the non-diabetic group, also referred to as the positive control (n = 8), that was orally administered *L. paraplantarum* BGCG11 for four weeks (10 mL; 10^8^ CFU/mL); (iii) D—the diabetic group (n = 8), that was injected with STZ (40 mg/kg; i.p.) for five consecutive days and left untreated throughout the four-week period; (iv) P/D/P—the pretreated diabetic group (n = 6), that received the BGCG11 strain at the same dose one week prior to the first STZ injection and kept receiving the BGCG11 strain for four weeks following the STZ treatment; (v) D/P—the post-treatment diabetic group (n = 6), which began on the last day of STZ administration and received the same daily dose of the BGCG11 strain for four weeks. Blood serum was obtained from the rats after an overnight fast following four weeks of diabetes.

### 4.4. Determination of General Indicators of Diabetes

After blood coagulation and centrifugation at 2000× *g* for 10 min, the serum was extracted. Blood glucose levels were measured in all experimental groups using a commercial kit (Gluco-quant Glucose/HK; Boehringer Mannheim, Mannheim, Germany) based on the hexokinase/G6P-DH enzymatic method at the start of the experiments, 24 h after the last dose of STZ was administered, and four weeks after the treatment. Hemoglobin (Hb) was determined according to Drabkin and Austin [89]. The colorimetric method was used to measure glycosylated hemoglobin (GlyHb), according to Parker et al. [90]. Serum zonulin level was determined by immunoblot analysis (goat anti-Human Haptoglobin H5015 (1:1000), Sigma-Aldrich, St. Louis, MO, USA). Immunoreactive bands were identified with a chemiluminescence detection system (Santa Cruz Biotechnology, Santa Cruz, CA, USA) and quantified using TotalLab electrophoresis software, ver. 1.10 (Phoretix, Newcastle-upon-Tyne, UK).

### 4.5. Histological Analysis and Immunostaining

All experimental groups’ pancreas and duodenum were removed, and they were all fixed in 10%-buffered formalin for histology and immunohistology analyses. All examined groups’ tissues were sectioned at a thickness of 5 µm after being blocked in paraffin for histological and immunohistochemical analysis (Leica DMLB, Wetzlar, Germany). Tissue sections for histological examination were coloured with hematoxylin and eosin (H&E) and observed under a light microscope. Sections of the pancreas and duodenum were stained with Masson trichrome stain for collagen-fibre detection [91]. For immunohistochemical analysis, deparaffinised sections were passed through xylene and rehydrated in sequentially graduated ethyl alcohol. Slides were incubated in 0.3%-hydrogen peroxide/methanol for 20 min to reduce nonspecific background staining due to endogenous peroxidase. After washing in PBS, the sections were treated with 0.01 M sodium citrate buffer at 98 °C for antigen retrieval. The cooled tissues were washed four times in PBS prior to application of the blocking serum for 5 min (0.05% Tween 20, 5% bovine serum albumin). The primary antibody was applied overnight at room temperature (RT). Primary polyclonal antibodies against insulin, glucagon, E-cadherin, fibronectin, and alpha-smooth muscle actin (alpha-SMA) (Santa Cruz Biotechnology, Santa Cruz, CA, USA) were diluted 1:50 in PBS with 2% dry skimmed milk. After washing in PBS, sections were incubated at room temperature for 1 h with secondary antibody horseradish peroxidase (1:100) (Santa Cruz Biotechnology, Santa Cruz, CA, USA). Tissues were incubated for 20 min at room temperature in a solution of 3,3′-diaminobenzidine (DAB). After washing with PBS, the tissues were counter-stained with haematoxylin and washed in water, and mounting media was applied to the coverslips. For the negative control, the primary antibody was not added to the sections.

### 4.6. Mucosal Fluid Transfer

Mucosal fluid transport was measured by the everted intestinal sac technique as described by Obembe et al. [92]. Briefly, the small intestine was removed, thoroughly washed in PBS, and cut into 10 cm segments for sac-making. Each segment was tied at one end and inverted, exposing the highly active mucosa. After blotting, segments with ligatures were weighed, filled with 1 mL of Krebs bicarbonate solution (118 mM NaCl, 4.7 mM KCl, 1.2 mM MgSO_4_, 1.25 mM CaCl_2_, 1.2 mM KH_2_PO_4_, 25 mM NaHCO_3_, 11 mM glucose) as the serosal fluid, and the free end was then tied. The filled sacs were weighed again, and the increase in weight was taken as the initial serosal volume (ISV). Krebs bicarbonate solution representing mucosal fluid (40 mL) was placed in an incubating flask, and the sacs were immersed and incubated for 30 min. The final serosal volume (FSV) in the sac was determined by weighing the blotted sac before and after draining its contents. The difference between the final weight of the empty sac and the initial weight of the empty sac represents gut fluid uptake (GFU). Mucosal fluid transfer (MFT) was then determined as FSV − ISV + GFU and expressed as µL/g sac/30 min.

### 4.7. DNA Extraction from Faeces

Faecal material from different diabetic rat groups (D (n = 3), P/D/P (n = 4), D/P (n = 4)) was collected at the beginning, at the moment of diabetes induction, and at the end of the experiments. It was kept at −80 °C, and metagenomic DNA was extracted from rat faeces using a commercially available kit, ZR Tissue DNA MiniPrep™ Kit (Zymo Research Corp., Irvine, CA, USA). The concentration of isolated DNA was measured on a BioSpec-nano spectrophotometer (Shimadzu, Columbia, MD, USA) and kept at −20 °C. All samples had a necessary concentration (≥12 ng/μL) and, in a final volume of 30 μL, were sent to Novogene Company (Beijing, China). The library was constructed, and the V3-V4 hypervariable region of the 16S rRNA amplicon was sequenced using an Illumina NovaSeq paired-end platform (Novogene Co., Ltd., Beijing, China). Quality control was performed at each step of the procedure. Each sample had a flattened refraction curve, which indicates sufficient depth during sequencing.

### 4.8. Data Display and Statistical Analysis

FLASH (V1.2.7 http://ccb.jhu.edu/software/FLASH/ (accessed on 18 September 2019)) was used to merge paired-end reads. Under particular filtering criteria according to the QIIME, quality filtering on the raw tags was carried out (V1.7.0 http://qiime.org/scripts/split_libraries_fastq.html (accessed on 18 September 2019)) in order to obtain high-quality clean tags. For species annotation of each representative sequence at each taxonomic rank, QIIME was performed (Version 1.7.0 http://qiime.org/scripts/assign_taxonomy.html (accessed on 18 September 2019)) using the Mothur method against the SSUrRNA database of SILVA Database. Alpha and beta diversity analyses were carried out using normalized OTU sets of data. Alpha diversity was expressed via four indices, including Shannon, Simpson, Observed-species, and Chao1. Beta diversity was visualized using Principal coordinates analysis (PCoA) calculated in QIIME (Version 1.7.0), displayed with R software (Version 1.4.1717), and expressed via Anosim and Adonis.

A one-way analysis of variance (ANOVA) followed by Tukey’s tests was used for multiple comparisons. Data subjected to statistical analysis were expressed as the means ± S.E.M. (standard error of the mean). Each experimental group consisted of from 6 to 8 rats. Statistical analysis and preparation of graphs were done in GraphPad Prism 8.4.3 software. Different letters indicate significant differences between treatments (*p* < 0.05) except for the presentation of relative taxa abundance. Data are presented as mean values ± the standard deviation from different experiments, except for Alpha diversity analysis and relative abundance of OTUs, which are expressed as median ± the standard deviation.

By utilizing the PICRUSt2 [93] pipeline, which is based on marker gene surveys and the MetaCyc pathways database with default settings, predictive metagenome functions at the peak of the disease were generated. Using the Galaxy framework, PICRUSt2 output was analyzed with LEfSe to reveal discriminative predicted paths between these groups; paths that met an LDA-significance threshold of >2 were then displayed.

## 5. Conclusions

Our results unequivocally showed the protective antidiabetic effects of *L. paraplantrum* BGCG11 in STZ-induced diabetic rats. Both the pre- and post-treatment of diabetic rats with the *L. paraplantrum* BGCG11 preserved the pancreatic β-cells and improved insulin production in the pancreas, preserved the integrity of the intestinal epithelium, reduced intestinal permeability, and decreased fibrotic process, impacting the diminishment of endotoxaemia, and positively affecting the microbiota composition. This study has shed light on the intricate effects of administering a particular strain of BGCG11 on the repair and function of damaged rats’ organs, which may help us understand how BGCG11 may benefit individuals with diabetes. Hence, in accordance with FAO/WHO criteria and EFSA recommendations, the next step will be to prove the safety and efficacy of *L. paraplantrum* BGCG11 strain as a potential probiotic in human clinical trials.

## Data Availability

The raw data supporting the conclusions of this article will be made available by the authors upon request.

## Supplementary Materials

The following supporting information can be downloaded at https://www.mdpi.com/article/10.3390/ijms25147697/s1.
