# Supplementary material for: Beneficial Effects of Probiotic Lactobacillus paraplantarum BGCG11 on Pancreatic and Duodenum Function in Diabetic Rats
Source: Int J Mol Sci. 2024 Jul 13;25(14):7697. doi: 10.3390/ijms25147697 (PMC11277547; doi:10.3390/ijms25147697)
Supplement: Supplementary file 1 [file ijms-25-07697-s001.zip › Supplementary figures.pdf]

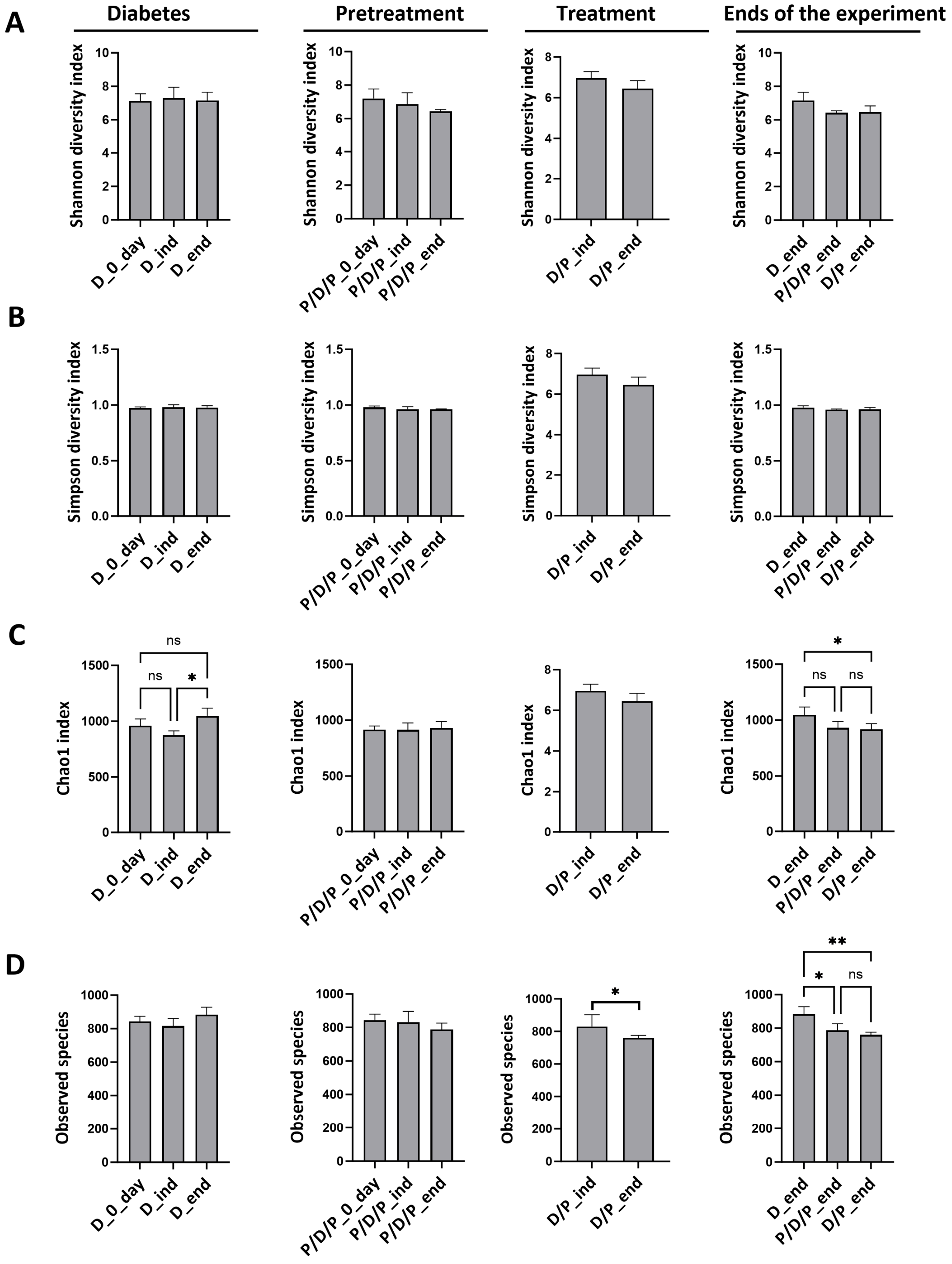

Figure S1. Alpha diversity as a parameter of biodiversity within the group, expressed via Shannon diversity index (A), Simpson diversity index (B), Chao 1 index (C) and Observed species (D), *represents significance (p<0.05), ** represents significance (p <0.01)

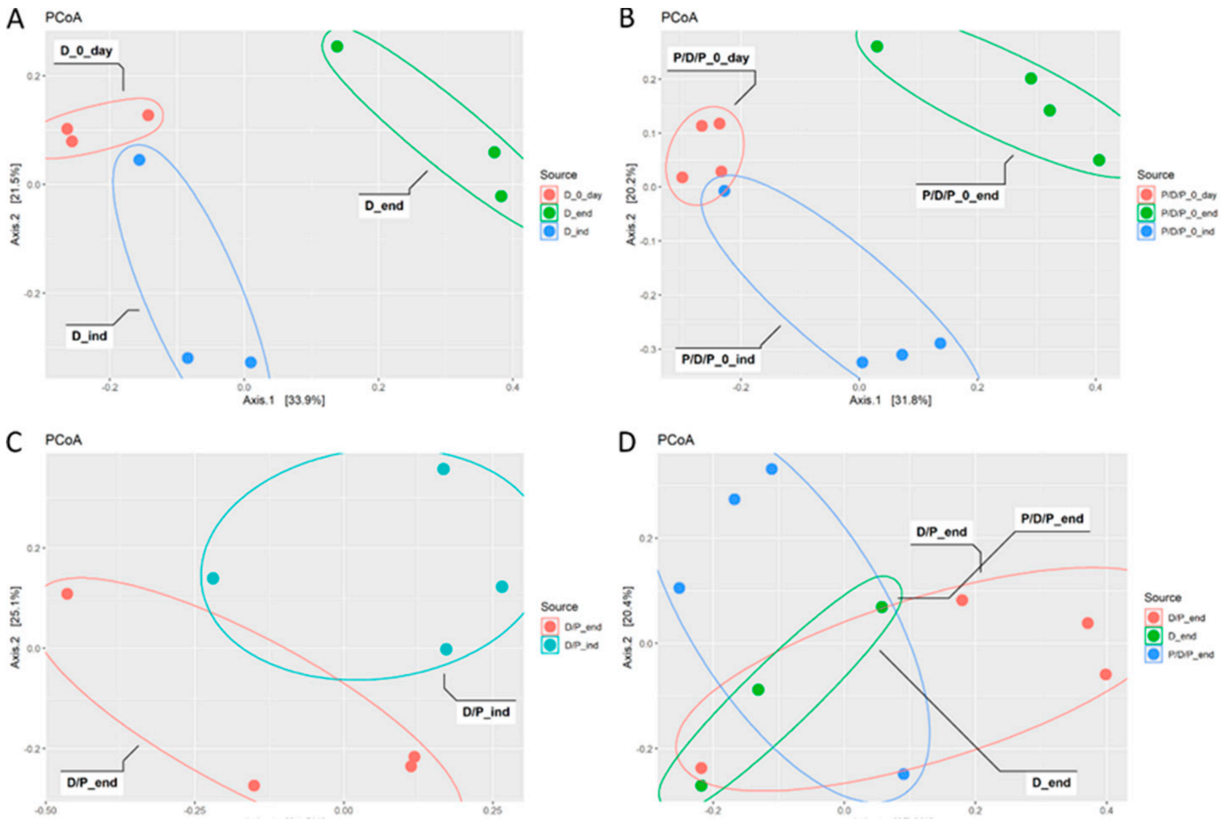

Figure S2. Beta diversity for comparison of different microbial communities, PCoA was used for its visuelization
